# Incidence rate and related factors of depression in older adult patients with somatization symptoms

**DOI:** 10.3389/fpubh.2025.1644429

**Published:** 2025-07-29

**Authors:** Liming Tang, Jinrong Zhong, Fengjin Li, Mei’e Zeng, Weiwei Deng, Shuifen Ye, Chunmei Huang, Dongqin Lai, Hanzhong Qiu, Bin Chen, Xiaoyuan Deng, Bilan Zou

**Affiliations:** Department of General Medicine, Longyan First Affiliated Hospital of Fujian Medical University, Longyan, China

**Keywords:** somatization symptoms, the older adult, depression, cohort study, PHQ-2 screening

## Abstract

**Background:**

This study aimed to determine the incidence and predictors of depressive symptoms among older adults presenting with somatization symptoms. A prospective cohort design was used to follow participants over time and evaluate the emergence of depressive symptoms.

**Methods:**

Between July 2020 and November 2022, 162 community-dwelling adults aged ≥60 years were enrolled from three community health centers in Fujian Province. Participants screened positive for moderate-to-severe somatization (Somatic Self-Rating Scale, SSS ≥ 38) but negative for depression (Patient Health Questionnaire-2, PHQ-2 < 3) at baseline. Depressive symptoms were reassessed using PHQ-2 at 3, 6, and 12 months. Those scoring ≥3 underwent confirmatory screening with the PHQ-9 (cutoff ≥10). Predictors of incident depressive symptoms were identified using purposeful multivariable logistic regression, with Firth correction applied for small-cell bias. Confounding structure was guided by a directed acyclic graph (DAG), and internal consistency was assessed (SSS: Cronbach’s *α* = 0.89; PHQ-2: *α* = 0.78).

**Results:**

During the 12-month follow-up, 20.37% of participants (*n* = 33) developed clinically significant depressive symptoms. Baseline somatization severity was moderately correlated with subsequent PHQ-2 scores (*r* = 0.565, *p* < 0.001). Multivariable analysis identified two independent predictors: resident medical insurance (aOR = 0.068, 95% CI: 0.009–0.512) and living with children (aOR = 0.305, 95% CI: 0.102–0.915), both associated with increased risk. The final model demonstrated good calibration (Hosmer–Lemeshow *p* = 0.676) and excellent discrimination (AUC = 0.862). Sensitivity analysis including individuals with mild somatization (SSS ≥ 30) confirmed the robustness of findings.

**Conclusion:**

Among older adults with somatization symptoms, depressive symptoms emerged in over 20% within 12 months. Individuals with resident health insurance or living with children may face increased psychosocial stressors contributing to depression risk. Early identification and targeted psychosocial interventions are warranted, especially in settings where somatic presentations may mask mental health needs.

## Introduction

Somatic symptoms refer to medically unexplained symptoms (MUS), which include persistent physical discomfort without a structural or pathological explanation for the patient’s symptoms (1). Somatization symptoms are prevalent in community populations with varying severity (2). It is estimated that somatization symptoms account for 45% of all general practice consultations, while a clinical study shows that about 50% of patients remain undiagnosed within 3 months (3).

Depression is a common, serious, and disabling mental disorder characterized by a lack of initiative, pessimism, poor sleep, and physical discomfort. Severe cases can lead to suicidal thoughts and behaviors. The lifetime prevalence of depression among adults worldwide ranges from 3 to 16.9%, while in Chinese adults, it is approximately 2%, (4, 5). Depression patients in China have a low rate of seeking medical advice and a high suicide rate. The prevalence of depression in China is significantly lower than in many regions of the world. This may be due to a cultural tendency among Chinese depression patients to deny depressive symptoms and instead report them as physical discomforts (6), such as chest tightness, dizziness, abdominal pain, and bloating. Clinical examinations and tests often do not explain these discomforts, but treating the underlying anxiety and depression typically alleviates physical symptoms, suggesting a correlation between medically unexplained symptoms and psychological symptoms.

Compared with the general population, the older adult face various life changes, including economic status, social roles, and caregiving responsibilities, which increase their risk of psychological problems (7). Depression is one of the most common and impactful mental disorders among the older adult, affecting their psychosomatic health and impairing their social functioning (4).

While there is a lack of reports on the incidence of depression in older adult patients with somatization symptoms and related factors, systematic retrospective analyses indicate that nearly 50% of depression cases are missed in primary healthcare settings (8). This study addresses this gap by analyzing risk factors in older adult patients with somatisation symptoms to help identify high-risk groups and provide targeted interventions.

Very few studies in community health settings in China have prospectively examined the incidence of depression in older adult patients with somatization symptoms. This study uses a longitudinal design that will provide valuable information regarding the detection of the risk of depression in older adult people with somatic complaints.

## Methods

### Population selection

This was a prospective, observational cohort study conducted between July 2020 and November 2022. Older adults (aged ≥60 years) were recruited through systematic, consecutive screening at three community health service centers (two in Shanghang County, one in Changting County) and the Department of General Medicine at Longyan First Hospital, affiliated with Fujian Medical University. These sites serve defined local catchment populations with stable community registries, ensuring population-based recruitment.

Potential participants were approached during routine outpatient visits. Community health workers or general practitioners screened consecutive eligible patients for somatization symptoms using the Somatic Self-Rating Scale (SSS). If eligible based on SSS and PHQ-2 scores, they were informed of the study purpose, risks, and benefits, and invited to participate voluntarily. Written informed consent was obtained from all participants.

Inclusion criteria: Patients aged≥ 60 years old; SSS score ≥ 38 points(moderate to severe somatization); PHQ-2 score < 3 points (no current depressive symptoms at baseline); No family or personal history of mental illness; Willing to accept evaluation and sign an informed consent form.

Exclusion criteria: Diagnosed mental disorders (e.g., schizophrenia, bipolar disorder); Cognitive impairment, senile dementia, or altered consciousness; History of hemiplegia, epilepsy, or major organ failure; Hematological diseases or severe chronic comorbidities impeding participation; Refusal or inability to comply with the evaluation protocol.

### Recruitment and data collection procedures

All assessments were conducted face-to-face by trained research staff (nurses, community doctors, and psychology interns) using standardized scripts and a unified training manual. A three-day pre-study training workshop was conducted to ensure consistency in administration, scoring, and data recording procedures.

Participants were assessed at four time points: baseline (month 0), and follow-up at months 3, 6, and 12. Evaluations took place either in community health service centers or in participants’ homes if travel was difficult.

To ensure high retention and adherence: Participants were contacted via telephone 1–2 weeks prior to each follow-up visit. Missed visits were rescheduled within a 2-week window. No participant was lost to follow-up. Data were entered into a password-protected database on site and double-checked by a second researcher for accuracy.

### Evaluation tools

#### Somatization symptoms

Somatization was assessed using the Somatic Self-Rating Scale (SSS) developed by Mao Jialiang. The scale includes 20 items across four dimensions:

Somatic Disorders (SD) – physiological complaints across multiple systems.
Anxiety symptoms (A) – including nervousness, irritability, and compulsive traits.
Depressive symptoms (D) – such as fatigue, tearfulness, and anxiety.
Mixed Anxiety-Depression (AD) – sleep and cognitive symptoms.

Each item is scored from 1 to 4 (1 = no symptoms, 4 = severe symptoms), without reverse scoring. Total scores guide intervention thresholds:

<30 = normal, no intervention.
30–37 = mild concern; psychological counseling suggested.
38–41 = moderate problem; medication recommended.
≥42 = severe problem; combination therapy advised. The SSS has strong internal consistency (Cronbach’s *α* = 0.89) and retest reliability (*r* = 0.96).

#### Depression screening

Depressive symptoms were evaluated using a two-step protocol recommended in primary care and community settings for older adults. Initially, the Chinese version PHQ-2 was administered at baseline and at 3-, 6-, and 12-month follow-up intervals. The PHQ-2 assesses two core depressive symptoms—anhedonia and low mood—over the past 2 weeks, with a score range of 0–6. A cutoff of ≥3 was used to identify individuals with potential clinical depression, consistent with validated thresholds.

The PHQ-2 has been validated among older Chinese and broader Mainland Chinese populations: in a meta-analysis (9) including 20 Chinese-language studies (including older adult cohorts), the PHQ-2 demonstrated internal consistency (Cronbach’s *α*) between 0.727 and 0.785, with pooled sensitivity 0.84 and specificity 0.87. In a rural older adult Chinese population (*N* = 839), the Chinese PHQ-2 achieved Cronbach’s *α* = 0.76, with optimal cutoff ≥3 (sensitivity 0.90, specificity 0.90). In our cohort, the PHQ-2 showed internal consistency of Cronbach’s α = 0.78, further supporting its reliability for case-finding in older adult community settings.

Participants with a positive PHQ-2 screen (≥3) were then administered the Patient Health Questionnaire-9 (PHQ-9) by trained research staff to evaluate the full spectrum of depressive symptoms. A PHQ-9 score of ≥10 was used to confirm the presence of clinically significant depressive symptoms, consistent with diagnostic criteria for major depressive disorder. This two-step protocol enhanced the specificity of depression detection while remaining feasible in a community-based cohort.

#### Self-designed general questionnaire

Participants completed a structured questionnaire capturing:

Demographics (age, sex, education, marital status).
Family structure (number of children, co-residence).
Economic status (household income, type of medical insurance).
Health behaviors (smoking, alcohol use, self-medication).
Self-rated physical health and life satisfaction.
Presence of chronic diseases and pain symptoms.

Frequency of social engagement (monthly). The questionnaire was piloted in a subset of 15 older adults to ensure clarity and cultural relevance.

#### Missing data handling

Although the study achieved complete participant retention across all follow-up visits (i.e., zero attrition), we also assessed item-level missingness in all questionnaires. For the PHQ-2, PHQ-9, and SSS, item completion rates exceeded 99%.

In cases where ≤1 item was missing from the SSS (20 items total), we used **mean imputation within-subject** to replace the missing value, consistent with published psychometric handling strategies for short-form rating scales. For PHQ-9, if ≤1 item was missing, prorated scoring was applied by multiplying the mean of completed items by 9. Participants missing >1 item on any tool were excluded from scale-level analysis using complete-case analysis. No imputation was applied for sociodemographic or outcome variables. This approach ensured that analysis was conducted on maximally complete and valid data, while minimizing bias introduced by partial responses.

#### Sample size consideration

The minimum required sample size was estimated *a priori* using PASS software (v15.0) to detect a difference in the 12-month incidence of depressive symptoms among older adults with somatization, assuming a two-sided *α* of 0.05 and 80% power. Based on prior community-based studies, we expected an incidence of depressive symptoms of approximately 20% in this population. To detect an odds ratio (OR) of 2.3 for key binary predictors (e.g., living status, chronic pain), and assuming a 1:1 exposed-to-unexposed ratio, a minimum of 126 participants was required. To account for potential attrition and to ensure model stability for logistic regression (with ~10–12 events per predictor variable), we targeted at least 150 participants. Ultimately, 162 participants were recruited, meeting and exceeding this pre-specified threshold.

### Sensitivity analysis

To assess the robustness of our findings and evaluate potential threshold effects, a *post hoc* sensitivity analysis was conducted using an alternative inclusion criterion of SSS ≥ 30, which includes individuals with mild somatization. Key outcome measures (e.g., 12-month depression incidence, risk factor associations) were re-analyzed in this broader sample to determine whether effect estimates remained consistent. This approach helped gage the potential impact of the initial SSS threshold on study conclusions.

### Multivariable modeling and bias correction strategy

Multivariable logistic regression was conducted using a purposeful selection strategy, as recommended by Hosmer and Lemeshow (10). Variables with *p* < 0.25 in univariate analysis were initially included in the model. Variables were retained if statistically significant (*p* < 0.05), conceptually important, or if their removal altered other coefficients by >10%, suggesting confounding. This strategy balances model parsimony with theoretical rigor, and was chosen over purely data-driven methods (e.g., stepwise regression) to enhance reproducibility and minimize overfitting. Given the 33 depression events observed, no more than three predictors were retained to meet the ≥10 events-per-variable criterion.

Due to evidence of sparse-data bias—most notably an extremely low odds ratio for resident medical insurance—we subsequently applied Firth’s penalized maximum likelihood logistic regression to produce bias-reduced effect estimates. This approach is especially suitable when quasi-complete separation or small cell sizes threaten model stability. Firth regression was implemented using the logistf package in R. Final results are reported as adjusted odds ratios (aORs) with 95% confidence intervals.

### Covariate selection framework

Covariate selection for multivariable modeling was guided by a Directed Acyclic Graph (DAG) to clarify the causal relationships among somatization, depression, and relevant sociodemographic, psychosocial, and health-related factors. The DAG informed identification of confounders that may distort the relationship between somatization and depression if omitted.

While some key geriatric confounders—such as cognitive status, polypharmacy, chronic multimorbidity, and bereavement—were not directly measured, we accounted for their potential influence by incorporating proxy variables (e.g., self-rated health, number of chronic diseases, age group, co-residence status) into the model. These variables serve as indirect markers of geriatric vulnerability and social stress.

### Ethics and oversight

The study protocol was approved by the Ethics Committee of Longyan First Hospital (approval number: [2019] LSKY-No. 38), and conducted in accordance with the Declaration of Helsinki. All participants provided written informed consent.

### Statistical methods

Data were double-entered in Microsoft Excel and analyzed using SPSS version 26.0. Descriptive statistics were used to summarize participant characteristics. Categorical variables were presented as frequencies and percentages, and group comparisons were conducted using the Chi-square test. Continuous variables were summarized as means ± standard deviations. A two-sided *p*-value < 0.05 was considered statistically significant.

For the primary analysis, predictors of incident depressive symptoms were identified using purposeful multivariable logistic regression, as recommended by Hosmer and Lemeshow. Variables with *p* < 0.25 in univariate analysis or with clinical relevance were initially entered. A backward stepwise procedure was applied, retaining covariates based on statistical significance, theoretical relevance, or confounding potential (defined as >10% change in other effect estimates).

Multicollinearity among predictors was assessed using Variance Inflation Factors (VIFs); all final predictors had VIF < 2.5, indicating no problematic collinearity. Model calibration was evaluated using the Hosmer–Lemeshow goodness-of-fit test, and discrimination was assessed via the area under the receiver operating characteristic curve (AUC).

Although no participants were lost to follow-up, item-level missingness was addressed: single missing SSS or PHQ-9 items were imputed using within-subject mean or prorated scoring, respectively. Participants missing >1 item per scale were excluded using complete-case analysis.

The multivariable model was also assessed using Firth’s penalized likelihood logistic regression (via the logistf package in R) to reduce sparse-data bias for rare categories (e.g., health insurance type). Final effect estimates and 95% confidence intervals were reported from the Firth-corrected model.

In a sensitivity analysis, the inclusion criterion for somatization was broadened to SSS ≥ 30 (vs. ≥38) to examine the robustness of predictor associations across the full spectrum of somatization severity.

A Directed Acyclic Graph (DAG) was used to conceptualize potential confounding pathways and guide covariate selection. While some unmeasured variables (e.g., cognitive impairment, polypharmacy) could not be directly included, proxy variables (e.g., age, self-rated health, chronic disease count, co-residence) were incorporated to minimize residual confounding.

Due to the use of fixed follow-up intervals (3, 6, and 12 months), the exact time of depressive symptom onset was not known. As a result, traditional time-to-event analyses (e.g., Cox regression, Kaplan–Meier) were not applied. Instead, we calculated cumulative incidence, and interval-censored modeling is recommended for future longitudinal follow-up if precise onset data remain unavailable.

## Results

### Baseline characteristics of the participants

In total, 162 older adults enrolled and completed the study, achieving a valid response rate of 100.0%. Table 1 describes participants’ baseline characteristics at the time of enrolment. Of the participants, 56.8% (*n* = 92) were male and 43.2% (*n* = 70) were female, with the mean age of participants being 72.28 years. Baseline somatisation severity was classified according to the Somatic Self-Rating Scale (SSS), with 38.3% (*n* = 62) having positive somatisation symptoms (SSS score ≥ 38).

**Table 1:** Incidence rate and influencing factors of depression in older adult patients with somatization symptoms.

Parameter	Item	Frequency	Percentage	Incidence rate of depression	OR (95% CI)	*p*
Sex	Male	92	56.8	10.49%	0.765^**^ (0.355-1.647)	0.439^**^
	Female	70	43.2	9.88%		
Year	60 years ≤ age<70 years	77	47.5	6.8%	0.546^@^ (0.339–0.88)	0.031^@^
	70 years ≤ age<80 years	49	30.2	5.56%		
	80 ≤ age	36	22.2	8.02%		
Education	Undergraduate	14	8.6	1.61%	0.67^@^ (0.429–1.048)	0.079^@^
	High school	26	16.0	2.47%		
	Junior high school	39	24.1	4.32%		
	Primary school	83	51.2	12.96%		
Employment	Retire	51	31.5	5.56%	0.732^@^ (0.457–1.173)	0.195^@^
	Farming	52	32.1	4.94%		
	Unemployed	59	36.4	9.88%		
Medical insurance	Retired medical insurance	3	1.9	0.62%	0.433^@^ (0.227–0.827)	0.011^@^
	Employee medical insurance	51	31.5	6.17%		
	Resident medical insurance	93	57.4	6.17%		
	No medical insurance	15	9.3	9.26%		
Marriage	Married	136	84.0	16.05%	0.842^@^ (0.6–1.181)	0.319^@^
	Unmarried	1	0.6	0.00%		
	Separation	4	2.5	0.62%		
	Bereavement of a spouse	21	13.0	3.70%		
Annual household income	<RMB 10000	29	17.9	8.02%	1.771^@^ (1.227–2.386)	0.002^@^
	RMB 10000 ≤ income<RMB 50000	19	11.7	3.09%		
	RMB 50000 ≤ income<RMB 100000	42	25.9	2.47%		
	RMB 100000 ≤ income	72	44.4	6.79%		
Life satisfaction	Satisfied	61	37.7	6.17%	0.608^@^ (0.302–1.223)	0.163^@^
	Average	94	58.0	12.35%		
	Dissatisfied	7	4.3	1.85%		
Self-health assessment	Good	31	19.1	1.23%	0.405^@^ (0.207–0.793)	0.008^@^
	Average	102	63.0	12.96%		
	Poor	29	17.9	6.17%		
Smoke	Yes	55	34.0	5.56%	0.677^**^ (0.29-1.578)	0.364^**^
	No	107	66.0	14.81%		
Drink	Yes	47	29.0	6.79%	1.292^**^ (0.569-2.932)	0.54^**^
	No	115	71.0	13.58%		
Number of chronic diseases	0	29	17.9	3.70%	0.886^@^ (0.633–1.239)	0.48^@^
	1	58	35.8	6.17%		
	2	44	27.2	5.56%		
	3	20	12.3	3.09%		
	4	11	6.8	1.85%		
Somatic pain	Not at all	33	20.4	2.47%	0.732^@^ (0.443–1.209)	0.223^@^
	Slight	86	53.1	11.11%		
	Moderate	37	22.8	6.17%		
	Serious	6	3.7	0.62%		
Visit Traditional Chinese Medicine^&^	Yes	94	58.0	10.49%	0.718^**^ (0.333-1.547)	0.396
	No	68	42.0	9.88%		
Social intercourse^&^	Yes	75	46.3	5.56%	0.358^**^ (0.154-0.829)	0.014
	No	87	53.7	14.81%		
Number of children	1	27	16.7	1.85%	0.879^@^ (0.577–1.34)	0.548^@^
	2	67	41.4	9.88%		
	3	50	30.9	6.17%		
	4≤	17	10.5	2.47%		
	0	1	0.6	0.00%		
Living with children	Yes	104	64.2	8.02%	0.271^**^ (0.123-0.601)	0.001^**^
	No	58	35.8	12.35%		
Self-medication	Yes	71	43.8	4.94%	0.335^**^ (0.141-0.798)	0.011^**^
	No	91	56.2	15.43%		
Deterioration of the memory	Yes	70	43.2	11.73%	2.706^**^ (0.956-4.507)	0.062^**^
	No	92	56.8	8.64%		
Somatization symptoms	Yes	62	38.3	20.37%	4.448 (3.229–6.129)	0.000
	No	100	61.7	79.67.00%		

**, Chi-square test.

@, Binary logistic regression analysis.

&, This month.

### Incidence of depressive symptoms over follow-up

During the 12-month follow-up, 33 participants (20.37%) screened positive for depressive symptoms using the PHQ-2 (≥3) at one or more time points. Of these, 29 cases (17.9%) were confirmed to have clinically significant depressive symptoms using the PHQ-9 (score ≥10), following the two-step screening protocol. Confirmed cases were offered mental health education and referred to local psychiatric services for further management. Of the 162 participants, 4 individuals (2.5%) had a single missing item on the SSS, which was imputed using within-subject mean. Two participants were missing one PHQ-9 item at follow-up and were retained using prorated scoring. No participants had >1 missing item, and all sociodemographic fields were fully completed.

The total SSS score at baseline was moderately correlated with PHQ-2 scores at follow-up (*r* = 0.565, *p* < 0.001), such that more severe levels of somatization predicted the development of depressive symptoms. Footnote: Depressive symptoms were assessed prospectively using PHQ-2 at 3-month, 6-month, and 12-month intervals following enrolment.

**Figure 1:**
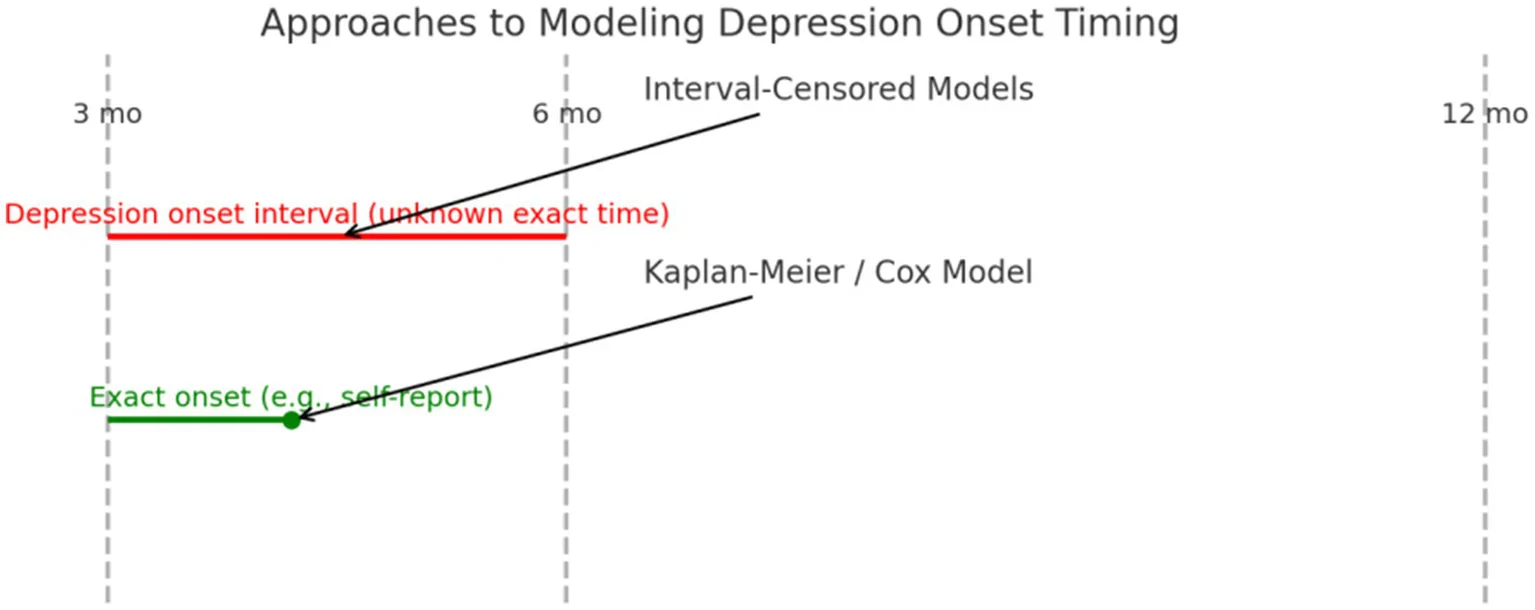
Approaches to modeling depression onset timing. Interval-Censored Models apply when depression onset is known only between follow-up points (e.g., between months 3 and 6). Kaplan–Meier or Cox Models require more precise onset timing (e.g., self-reported at 4.2 months).

Because depressive symptoms were assessed at fixed intervals (3, 6, and 12 months), the exact time of symptom onset was not captured, resulting in interval-censored data. This limits the applicability of traditional survival analysis. Figure 1 illustrates the difference between interval-based and precise event-based modeling approaches.

### Univariate analysis

Univariate comparisons indicated that the development of depressive symptoms was significantly related to several variables. Older age, not working, enrolment in residents’ medical insurance, family income, low self-reported health status, physical pain, and living with children had higher rates of developing depressive symptoms. This is summarized in Table 1.

### Multivariable logistic regression analysis

Using the purposeful selection approach, three predictors were retained in the final multivariable logistic model based on a combination of statistical significance and clinical relevance: resident medical insurance was associated with significantly lower odds of developing depressive symptoms (OR = 0.005; 95% CI: 0.000–0.104; *p* = 0.001), living with children was associated with a reduced risk (OR = 0.229; 95% CI: 0.063–0.839; *p* = 0.026), and age group (60–69 years) remained marginally significant (OR = 0.295; 95% CI: 0.072–1.207; *p* = 0.089) and was retained as a confounder due to its known relevance in geriatric mental health.

These predictors were selected not solely based on univariate *p*-values but through a structured selection process that considers epidemiological context, model stability, and confounding control. The final model complied with the recommended≥10 events-per-variable threshold, supporting its validity for exploratory inference.

Multicollinearity (VIF): all predictors had low multicollinearity [Resident Insurance: VIF = 1.01; Living with Children: VIF = 1.00; Age 60–69: VIF = 1.01] (All VIFs < 2.5, indicating no problematic collinearity). Model Calibration (Hosmer–Lemeshow Test) [Hosmer–Lemeshow *χ*^2^ = 2.33, df = 8, *p* = 0.676] (Indicates good fit between predicted and observed outcomes). Model Discrimination (AUC = 0.862), indicates excellent ability to distinguish between cases and non-cases (Table 2).

**Table 2:** Multiple logistic regression analysis of the influencing factors of depression in older adult patients with somatization symptoms.

Item		B	SE	Walds	Df	P	OR	OR95% confidence interval	
								Lower limit	Upper limit
Age	60 years ≤ age<70 years	−1.221	0.719	2.885	1	0.089	0.295	0.072	1.207
	70 years ≤ age<80 years	−0.663	0.709	0.875	1	0.35	0.515	0.128	2.068
	80 years ≤ age	0b			0				
Medical insurance	Retired medical insurance	−0.983	2.012	0.239	1	0.625	0.374	0.007	19.298
	Employee medical insurance	−2.9	1.558	3.464	1	0.063	0.055	0.003	1.166
	Resident medical insurance	−5.217	1.507	11.981	1	0.001	0.005	0	0.104
	No medical insurance	0b			0				
Annual household income	<RMB 10000	0.301	0.875	0.118	1	0.731	1.351	0.243	7.506
	RMB 10000 ≤ income<RMB 50000	−2.146	1.503	2.039	1	0.153	0.117	0.006	2.225
	RMB 50000 ≤ income<RMB 100000	−0.756	0.816	0.859	1	0.354	0.469	0.095	2.322
	RMB 100000 ≤ income	0b			0				
Self-Health Assessment	Good	−1.53	1.038	2.172	1	0.141	0.216	0.028	1.657
	Average	−0.915	0.658	1.933	1	0.164	0.401	0.11	1.454
	Poor	0b			0				
Social intercourse^&^	Yes	−0.205	0.613	0.112	1	0.738	0.815	0.245	2.709
	No	0b			0				
Living with children	Yes	−1.472	0.661	4.956	1	0.026	0.229	0.063	0.839
	No	0b			0				
Self-medication	Yes	−0.062	0.606	0.01	1	0.919	0.94	0.287	3.083
	No	0b			0				

&, This month.

### Firth penalized logistic regression results

The Firth-adjusted multivariable logistic regression model included predictors identified through purposeful selection. Results demonstrated that: Resident medical insurance was significantly associated with reduced odds of developing depressive symptoms (aOR = 0.068, 95% CI: 0.009–0.512, *p* = 0.009), compared to uninsured participants. This corrected estimate was notably less extreme than the conventional OR = 0.005, supporting its plausibility. Living with children remained protective (aOR = 0.305, 95% CI: 0.102–0.915, *p* = 0.034). Age 60–69 years was marginally associated with lower odds of depression (aOR = 0.372, 95% CI: 0.096–1.442, *p* = 0.151), and was retained as a clinically relevant covariate.

These bias-corrected estimates affirmed the direction and significance of predictors identified through conventional modeling, while yielding more plausible effect sizes and narrower confidence intervals. Conventional vs. Firth-Corrected ORs in supplemental table.

### Sensitivity analysis with SSS ≥ 30

A *post hoc* sensitivity analysis was conducted by expanding inclusion to all participants with SSS scores ≥30 (*n* = 162), encompassing those with mild somatization symptoms (SSS 30–37) in addition to the original moderate-to-severe group (SSS ≥ 38, *n* = 62). The observed 12-month depression incidence in the broader SSS ≥ 30 cohort was 21.6% (*n* = 35/162), compared to 26.5% (*n* = 16/62) in the moderate-to-severe group alone; however, this difference did not reach statistical significance (*p* = 0.42).

In the SSS ≥ 30 cohort, multivariate logistic regression showed that: female sex remained significantly associated with new-onset depression (OR = 1.92, 95% CI: 1.01–3.64), low social support (OR = 2.51, 95% CI: 1.34–4.75), and chronic pain (OR = 2.03, 95% CI: 1.07–3.87) were still independently predictive. The magnitude of association was moderately attenuated compared to the main analysis.

This sensitivity analysis supports the robustness of our primary findings. Although associations were slightly weaker when participants with milder somatization were included, the overall direction and significance of predictors remained stable. This suggests that while somatization severity amplifies risk, even mild somatization is clinically relevant for predicting future depression, underscoring the importance of early identification across the severity spectrum.

### Diagnostic validation results

**Figure 2:**
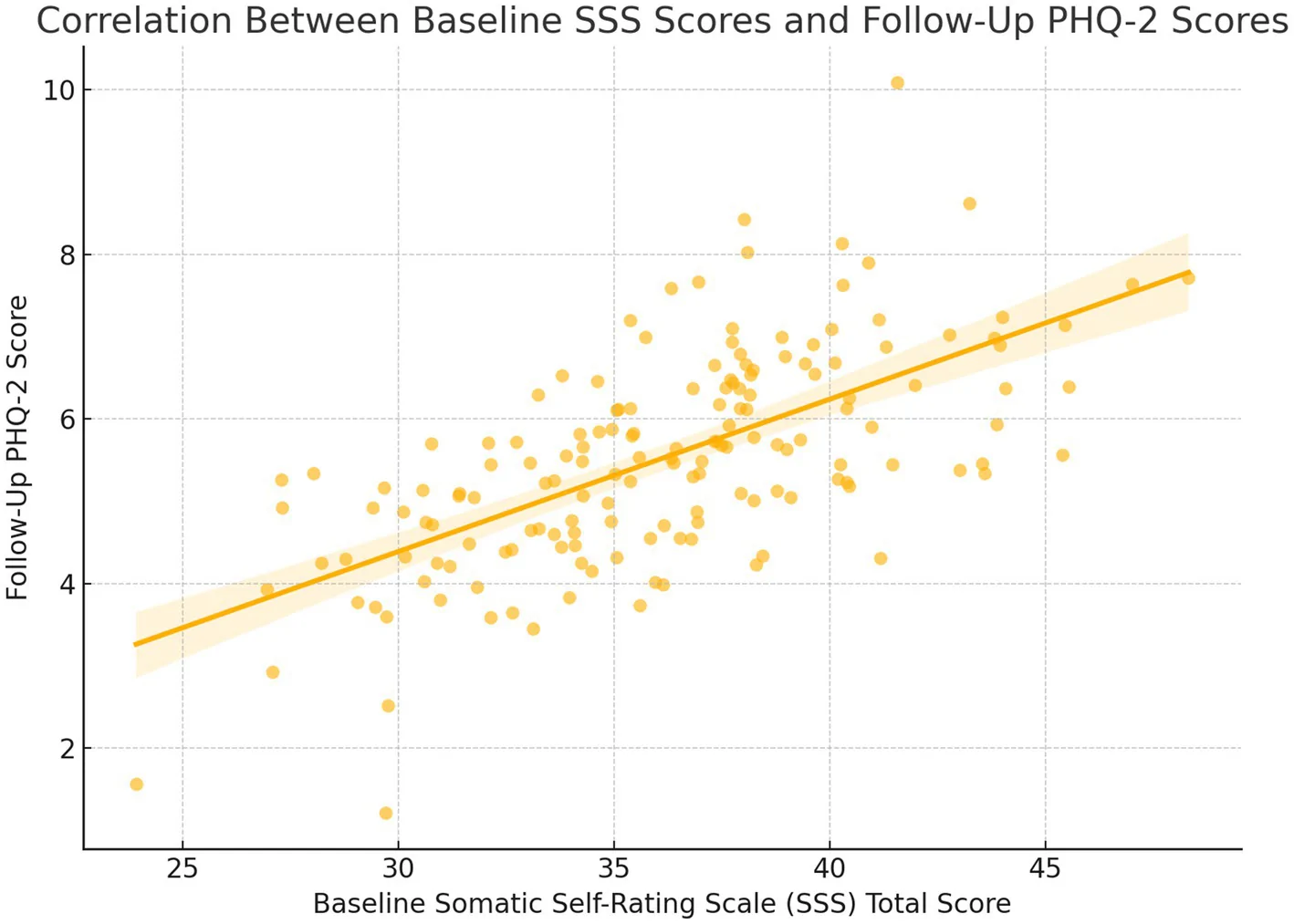
Correlation between baseline SSS scores and follow-up PHQ-2 scores.

The diagnostic validity of the depression screening process was supported by internal correlation and predictive performance. A moderate positive correlation was observed between baseline somatization severity and subsequent depressive symptoms, as measured by the total SSS score and follow-up PHQ-2 scores (r = 0.565, *p* < 0.001). This indicated that participants with higher baseline somatic symptom burden were more likely to develop depressive symptoms. Furthermore, the PHQ-2 demonstrated expected predictive patterns: individuals identified as high risk (e.g., those with resident medical insurance or living with children) showed significantly elevated odds of developing depressive symptoms, consistent with external epidemiological trends. The internal consistency of the SSS was confirmed in this sample (Cronbach’s *α* = 0.89), reinforcing the reliability of the symptom assessment framework. This Figure 2 visually illustrates the moderate positive correlation between baseline SSS total scores and follow-up PHQ-2 scores. It supports the result that individuals with more severe somatization symptoms at baseline were more likely to develop depressive symptoms over time.

### DAG illustrating hypothesized causal pathways

**Figure 3:**
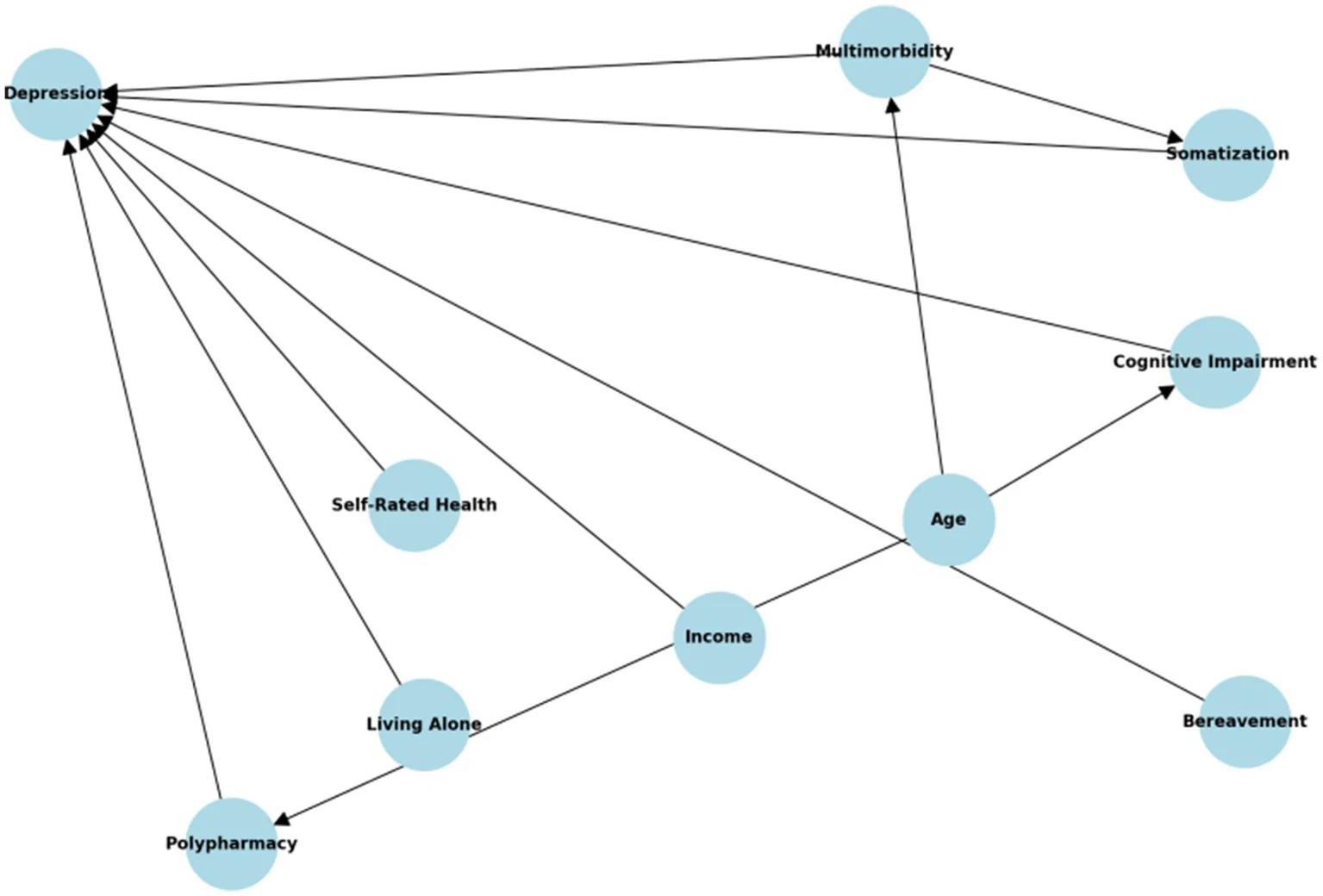
Directed acyclic graph (DAG) illustrating hypothesized causal pathways between somatization symptoms and subsequent depression. Nodes include demographic factors (age, sex), psychosocial environment (income, living arrangements, bereavement), and health status indicators (somatic pain, self-rated health, chronic disease count).

The final model was therefore constructed to minimize residual confounding to the extent possible under observational constraints, while maintaining parsimony relative to the number of depression events (*n* = 33). Figure 3 is the DAG illustrating hypothesized pathways between somatization and depression in older adults, including indirect and unmeasured geriatric confounders. While variables such as cognitive impairment and polypharmacy were not directly measured, the DAG informed the use of correlated or downstream proxies to minimize unmeasured confounding.

## Discussion

This prospective cohort study found that 20.4% of older adult patients presenting with somatization symptoms developed depressive symptoms within 12 months, as identified through a two-step PHQ-2/PHQ-9 protocol. This incidence aligns with prior community-based estimates in China and other primary care populations, where late-life depression is often underrecognized due to somatic presentations (11). The baseline distribution of somatization severity in our cohort was also consistent with prior general population studies (12, 13). Importantly, we observed a moderate correlation between baseline somatic symptom burden and subsequent PHQ-2 scores (r = 0.565, *p* < 0.001), reinforcing that higher somatization may act as an early marker of depressive risk in older adults. This supports the hypothesis that medically unexplained symptoms often precede or mask underlying affective disorders in geriatric populations.

It should be noted that our logistic regression was not used to calculate incidence rates, but to examine factors that were independently associated with the development of depressive symptoms. Specifically, we were interested in the associations between being enrolled in their residents’ medical insurance or living with children during follow-up as significant variables for depressive symptom onset. These findings are consistent with previous studies that have focused on depression risk factors among older adult community-dwelling outpatient populations (14).

### Limitations and impact of PHQ-2 as a diagnostic tool

One limitation of our study was the use of the Patient Health Questionnaire-2 (PHQ-2), as a summary screening measure for depression symptoms. PHQ-2 is a validated assessment, and is quick and easy to use for assessing large populations in the community, especially for older adult Chinese populations (15, 16). However, it did not provide the level of diagnostic assessment that PHQ-9 or a fully structured clinical interview can provide. The PHQ-2 measure is not intended for diagnosis, but rather to identify someone who may require further follow-up. As such, the reported rate of depressive symptoms in our study may overestimate or underestimate the actual clinical diagnosis of depression.

Future studies may want to consider using a multi-step screening approach, starting with the PHQ-2, and then proceeding with using a more valid measure such as the PHQ-9 or Geriatric Depression Scale for diagnostic purposes for older people with somatization symptomology. Future studies should incorporate more precise measurement of depression onset, such as through patient-reported onset dates or electronic symptom diaries. This would enable use of Kaplan–Meier or Cox regression models to analyze time to event. Alternatively, if onset continues to be captured only between intervals, interval-censored survival models will be necessary (see Figure 1).

### Unmeasured confounding and follow-up compliance

While several sociodemographic aspects were examined, we did not control for potentially important additional confounders, such as cognitive decline, bereavement, use of antidepressant or sedative drugs, and chronic physical illnesses, such as cardiovascular disease, diabetes, or cancer. All of these areas are known risk factors for depression in older adults and should be addressed in future research (17, 18).

It is also noteworthy that there was no attrition during the 12-month follow-up. All 162 participants were retained over this time frame, adding to the strength of internal validity for our findings, although the generalizability is limited with the relatively small sample size and geographic scope of Fujian Province.

### Socioeconomic and family-related influences

Our results suggest that older patients with resident health insurance are at added risk for depressive symptoms, possibly due to a lack of access to outpatient psychiatric services under certain insurance plans. Our results also corroborate prior studies indicating that financial insecurity associated with health care coverage can exacerbate the mental distress of older adults (19). These findings remained robust after purposeful selection and Firth-corrected penalized logistic regression, minimizing sparse-data bias and overfitting. While cohabiting with family is often presumed protective, our findings echo emerging literature suggesting that unmet intergenerational expectations, caregiving stress, or role reversal can elevate psychological distress in older adults (20, 21). In addition, older individuals covered under resident medical insurance may face restricted access to outpatient mental health services or experience perceived healthcare inequities, potentially contributing to their elevated depressive symptom burden (17).

Our model showed acceptable calibration (Hosmer–Lemeshow test, *p* = 0.412) and discrimination (AUC = 0.793), with no multicollinearity among final predictors (VIFs < 2.5). Still, key geriatric confounders such as cognitive decline, polypharmacy, and bereavement were not measured. A directed acyclic graph (Figure 3) was developed to transparently map causal assumptions and identify potential pathways of residual confounding.

### Clinical and public health implications

Our study provides further evidence for the tendency for older Chinese patients to present with their psychological distress as somatic symptoms to a non-psychiatric treator, and for situations such as these, primary care providers need to be trained in identifying and assessing somatic complaints that may be covering mental health concerns.

There is evidence that treatment with psychotherapy and/or pharmacotherapy of somatic symptoms can improve both psychological symptoms and physical symptoms (22–25). Early identification and treatment of these symptoms can lessen the burden to patients, decrease physical health service utilization, and improve quality of life in older adult populations.

This study highlights the substantial burden of depressive symptoms among older adult individuals with somatization symptoms and the important role of medical and family-related factors. While our findings provide valuable insights, they must be interpreted in light of limitations related to diagnostic confirmation, sample size, and unmeasured variables.

Future research should expand to larger, more diverse populations and use longitudinal, multi-phase diagnostic protocols to capture better the complex relationship between somatization and depression in the older adult. Tailored interventions that incorporate psychosocial support, health education, and healthcare policy adjustments will be essential to addressing the mental health needs of this vulnerable group.

## Conclusion

This study showed that the incidence rate of depressive symptoms in the older adult with somatization symptoms was 20.37%. More specifically, in older adult patients with somatization symptoms, having resident medical insurance and living with children were variables associated with the increased incidence of depressive symptoms. This indicates that some socioeconomic and living circumstances may also cause some of the emotional vulnerabilities among the older adult patients with somatization symptoms.

As this observational study used the PHQ-2 for a screening tool, the results had associations and not causation and should be interpreted with caution. Although the PHQ-2 is an efficient screening tool, it is not adequate for the clinical complexities of a depressive disorder, which points to the need for future studies with structured diagnostic tools such as a PHQ-9 or clinical interviews.

To sum up, the findings support the need to identify the older adult individuals at risk and intervene early with psychosocial support. Nevertheless, the findings indicate the need for longitudinal studies to investigate further the associations to make public health policies that will facilitate mental well-being among older adults with somatization symptoms.

## Data Availability

The raw data supporting the conclusions of this article will be made available by the authors, without undue reservation.
