# Correction: Incidence rate and related factors of depression in older adult patients with somatization symptoms

**DOI:** 10.3389/fpubh.2026.2012410

**Published:** 2026-10-23

**Authors:** Liming Tang, Jinrong Zhong, Fengjin Li, Mei'e Zeng, Weiwei Deng, Shuifen Ye, Chunmei Huang, Dongqin Lai, Hanzhong Qiu, Bin Chen, Xiaoyuan Deng, Bilan Zou

**Affiliations:** Department of General Medicine, Longyan First Affiliated Hospital of Fujian Medical University, Longyan, China

**Keywords:** somatization symptoms, the older adult, depression, cohort study, PHQ-2 screening

In the published article, there were errors in References 15–18. During reference management and formatting, incorrect bibliographic information was inadvertently included for these references. The correct references are as follows:

15. Chen S, Chiu H, Xu B, Ma Y, Jin T, Wu M, et al. Reliability and validity of the PHQ-9 for screening late-life depression in Chinese primary care. *Int J Geriatr Psychiatry*. (2010) 25:1127–33. doi: 10.1002/gps.2442

16. Li C, Friedman B, Conwell Y, Fiscella K. Validity of the patient health questionnaire 2 (PHQ-2) in identifying major depression in older people. *J Am Geriatr Soc*. (2007) 55:596–602. doi: 10.1111/j.1532-5415.2007.01103.x

17. Yan R, Li L, Duan X, Zhao J. Association of depressive symptoms with health service use and catastrophic health expenditure among middle-aged and older Chinese adults: analysis of population-based panel data. *J Am Med Dir Assoc*. (2023) 24:664–71.e7. doi: 10.1016/j.jamda.2022.11.018

18. Alexopoulos GS. Depression in the elderly. *Lancet*. (2005) 365:1961–70. doi: 10.1016/S0140-6736(05)66665-2

The corresponding in-text citations and reference numbering have been corrected accordingly where necessary.

The original version of this article has been updated

